# Dupilumab‐induced skin‐associated side effects in patients with chronic rhinosinusitis with nasal polyposis

**DOI:** 10.1111/1346-8138.16595

**Published:** 2022-09-30

**Authors:** David Chromy, Tina Bartosik, Faris F. Brkic, Tamara Quint, Aldine Tu, Julia Eckl‐Dorna, Sven Schneider, Christine Bangert

**Affiliations:** ^1^ Department of Dermatology Medical University of Vienna Vienna Austria; ^2^ Department of Otorhinolaryngology, Head and Neck Surgery Medical University of Vienna Vienna Austria

**Keywords:** CRSwNP, Dupilumab, nasal polyposis, psoriasiform dermatitis, rosacea‐like folliculitis

## Abstract

Chronic rhinosinusitis with nasal polyposis (CRSwNP) is a typical type‐2 inflammation involving T‐helper type‐2 cells and impairing quality of life due to nasal obstruction, discharge and reduced sense of smell. Recently, the anti‐IL4Rα antibody dupilumab was approved for CRSwNP. While dermatologic side effects in patients treated with dupilumab for atopic dermatitis are frequently observed, there is limited knowledge about these effects in patients with CRSwNP. We aimed to investigate frequency and characteristics of dermatologic side effects following initiation of dupilumab treatment in a cohort of Austrian CRSwNP patients. Therefore, CRSwNP patients presenting at the Department of Otorhinolaryngology, Head and Neck Surgery at the Vienna General Hospital were retrospectively evaluated for newly developed skin eruptions while under dupilumab treatment. Incidence was calculated and details on clinical symptoms were collected. One hundred and ninety‐two CRSwNP patients receiving dupilumab treatment were included, comprising a cumulative follow‐up of 89.65 years (median: 5.5, IQR: 5.9). We observed dermatologic side effects in four patients starting at a median time of 15.5 (range 4–23) weeks after dupilumab initiation corresponding to an incidence‐rate of 4.46 (95%‐confidence interval 1.39–11.23) events per 100 patient‐years follow‐up. The majority (75%, 3/4) of affected patients developed psoriasis‐like dermatitis, whereas one individual experienced rosacea‐like folliculitis and alopecia areata. While dupilumab dosing was reduced in 3/4 CRSwNP patients, one patient completely stopped dupilumab therapy. Our study provides the first comprehensive evaluation of both frequency and characteristics of dermatologic side effects caused by dupilumab in CRSwNP patients. All affected patients developed Th1‐inflammatory associated skin disorders – previously observed only in individuals with prior affections of the skin (i.e. atopic dermatitis). Thus, individuals receiving dupilumab for CRSwNP may develop novel symptoms that require interdisciplinary management. Future studies on dupilumab in a real‐world setting will be required to further explore its spectrum of side effects.

## 1 INTRODUCTION

The estimated prevalence of chronic rhinosinusitis with nasal polyposis (CRSwNP) in Western countries is 1.95%–4%.^1^, ^2^, ^3^ CRSwNP is characterized by hyperplasia of the sinonasal mucosa that leads to nasal obstruction, nasal discharge, impaired olfactory performances and poor quality of life. Immunologically, CRSwNP is associated with a type‐2 inflammation involving T‐helper type‐2 cells (Th2), type‐2 innate lymphoid cells and interleukin (IL)‐4, IL‐5, IL‐13 signaling and an infiltrate of mast cells, eosinophils and basophils within the polyps.^4^ Dupilumab, a monoclonal antibody blocking the IL‐4/IL‐13 receptor component IL‐4Rα, has been recently approved for the treatment of various type‐2 inflammatory diseases including moderate‐to‐severe atopic dermatitis (AD), chronic rhinosinusitis and moderate‐to‐severe asthma.^5^, ^6^

While dupilumab is generally well tolerated, typical adverse events in patients with AD comprise ocular complications, dermatologic eruptions (e.g. psoriasiform dermatitis, alopecia areata) and rare cases of other diseases.^6^, ^7^, ^8^ A recent report based on the FDA's ‘Adverse Event Reporting System’ found significantly less dupilumab‐related adverse events in patients with CRSwNP as compared to those with AD or asthma.^9^ While this study could not further specify adverse skin reactions, series of case reports are describing onset of psoriasiform dermatitis following dupilumab initiation in AD patients.^10^, ^11^ Blocking IL4Rα potentially leads to conversion from Th2‐ to Th1 or Th17‐dominated immune pathways resulting in psoriasis‐ or rosacea‐like skin eruptions.^12^ Whether this immunologic phenomenon is limited to patients with prior affection of the skin (i.e. AD) remained unanswered. To gain information on dermatologic side effects of dupilumab in CRSwNP patients in a real‐world setting, we evaluated an Austrian cohort of CRSwNP patients and described the frequency and nature of newly developed skin eruptions.

## 2 MATERIALS AND METHODS

### 2.1 Study design

All patients diagnosed with CRSwNP and receiving at least one dose of dupilumab at the Department of Otorhinolaryngology, Head and Neck Surgery at the Vienna General Hospital between February 2020 and January 2022 were retrospectively evaluated. Onset‐date of CRSwNP, data on comorbidities as well as new onset of skin lesions were collected. When patients experienced a new onset of skin disease while under dupilumab treatment, they were referred to the department of dermatology for further assessments and diagnosis. If onset of skin eruptions occurred exclusively after dupilumab initiation, affected patients were considered to have a dermatologic side effect. Incidence rate and 95% confidence interval (95%CI) of dermatologic side effects during follow‐up was calculated.

### 2.2 Ethics

This study was conducted in accordance with the 1964 Declaration of Helsinki and had been approved by the ethics committee of the Medical University of Vienna (No. 2222/2021). Due to the retrospective design, the need for an informed consent had been waived. The patients pictured in this manuscript have given written informed consent to publication of their case details.

## 3 RESULTS

**FIGURE 1:**
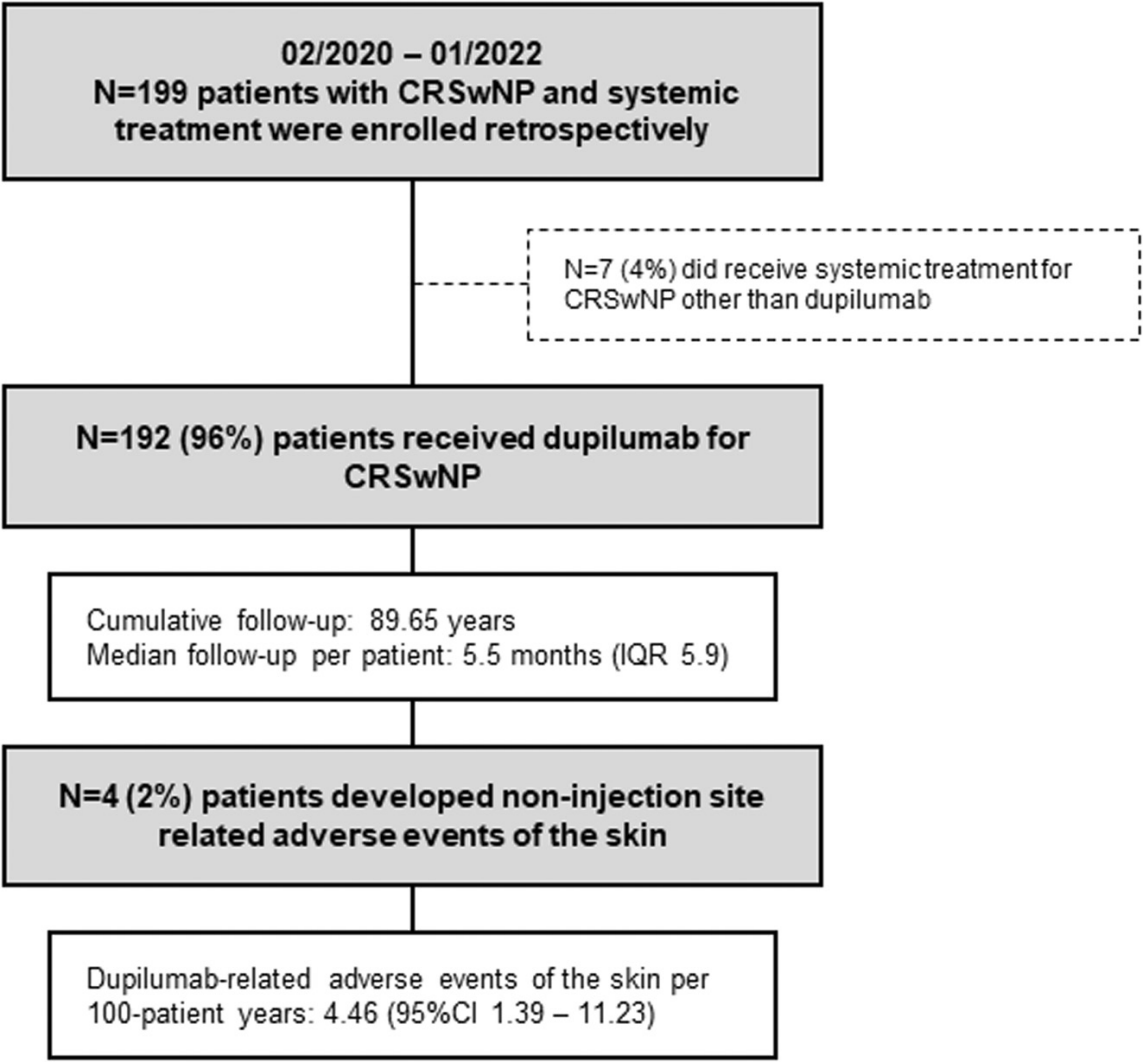
Patient consort diagram. Abbreviations: CI, confidence interval; CRSwNP, chronic rhinosinusitis with nasal polyposis; IQR, interquartile ratio

Between February 2020 and January 2022, 199 patients (male, *N* = 122; female, *N* = 77) received systemic treatment for CRSwNP of which 96% (192/199) were started on dupilumab (Figure 1). Sixty‐five percent (125/192) showed comorbid asthma, 54% (103/192) had one or more allergies in their medical history and 35% (68/192) were diagnosed with NSAID‐exacerbated respiratory disease (Table S1).

Four skin‐related adverse events were recorded during a cumulative follow‐up of 89.65 years resulting in an incidence‐rate of 4.46 (95%‐confidence interval 1.39–11.23) events per 100 patient‐years (PY). The median follow‐up was 5.5 months (IQR 5.9) and the median time from dupilumab initiation to onset of dermatologic side effects was 15.5 (range 4–23) weeks.

### 3.1 In‐depth analysis of patients with dermatologic side effects

**FIGURE 2:**
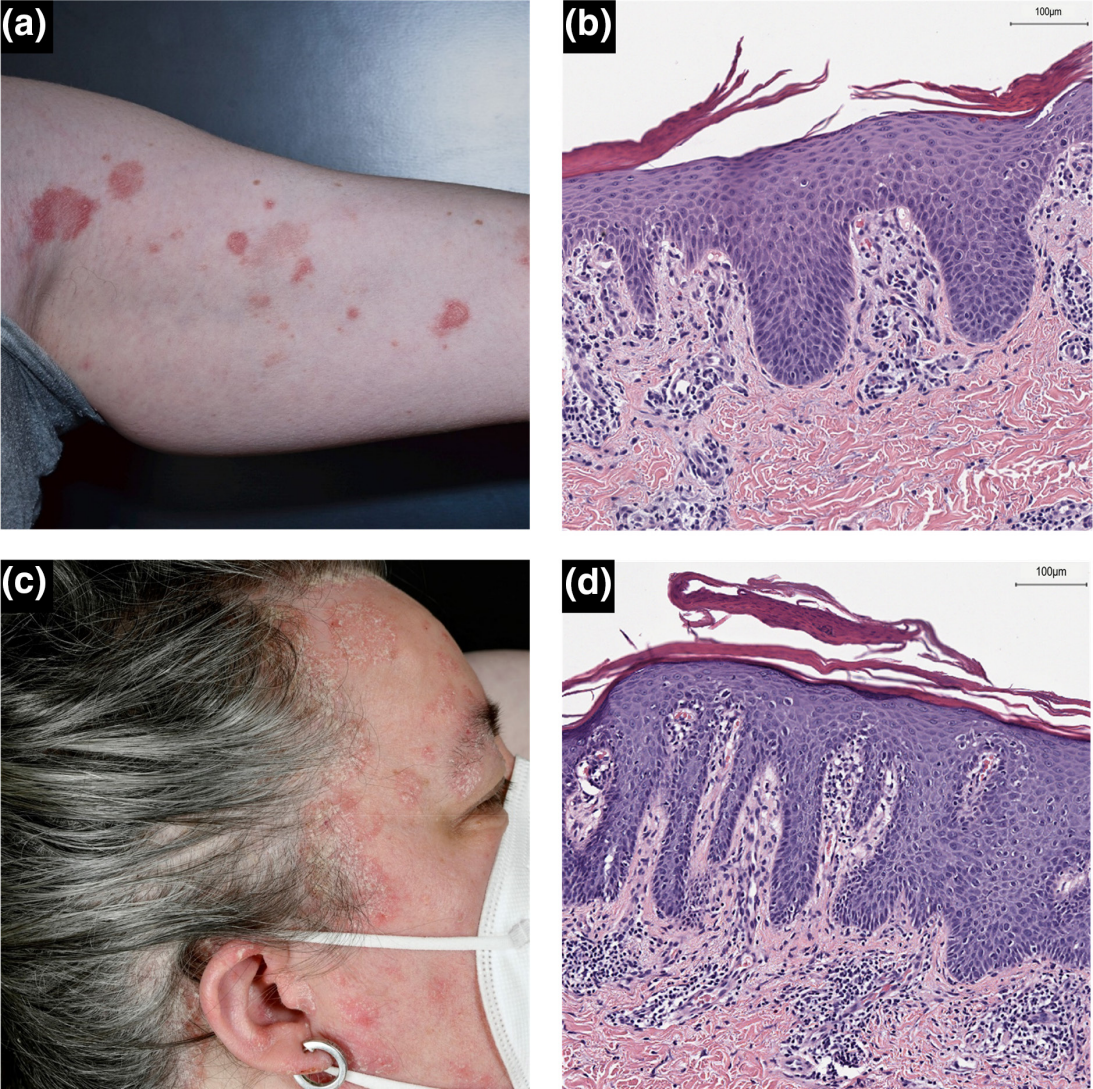
In patient A, disseminated erythematous plaques with slight, but definite scaling were found on both arms (left arm, (a)) and upper thighs. Punch biopsy revealed a lamellar stratum corneum, parakeratosis and spongiosis with lymphocytic infiltrate, psoriasiform acanthosis with thinning of the granular cell layer and dilated blood vessels and erythrocytic extravasates in the papillary dermis reaching up to dermal‐epidermal junction zone in the hematoxylin and eosin staining (b). In patient B, erythematous plaques with distinct white scaling occurred on all extremities, on the scalp and around the face and ears (c). Histologic examination showed clear signs of psoriasiform acanthosis, parakeratosis, neutrophil granulocytes in the stratum corneum and papillomatosis with dilated capillaries (d).

All patients showed a significant improvement of CRSwNP upon dupilumab initiation (Table 1). Patient A presented with multiple nummular erythematous lesions with adherent scaling on both upper arms and thighs after the second dose of dupilumab (Figure 2a). Histological examination revealed a psoriasiform inflammation (Figure 2b). Treatment was maintained and psoriatic lesions were treated with topical betamethasone/calcipotriol. Due to another psoriatic flare dupilumab intervals were extended to 4 weeks leading to stable skin disease requiring only topical corticosteroids treatment. Patient B developed erythematous lesions with scaling in the facial/temporal area, the scalp, trunk and extremities after 14 weeks of dupilumab (Figure 2c, Figure S1a). Clinical and histological features were specific for psoriasis (Figure 2d). As topical treatment with betamethasone/calcipotriol and dupilumab dose reduction was not effective, dupilumab was discontinued. In order to maintain therapy for CRSwNP omalizumab was initiated instead. Subsequently, all lesions resolved within 4 weeks. Patient C developed pustules and an itching/burning sensation of the facial skin as well as an alopecic area of the beard after 17 and 25 weeks after dupilumab initiation, respectively (Figure S1b,c,d). Dupilumab intervals were extended and rosacea‐like skin lesions were effectively treated using recurrent topical/systemic cycles with metronidazole, doxycycline and ivermectin. Patient D developed disseminated guttate psoriasis‐like lesions after 23 weeks. However, upon presentation at our clinic, topical corticosteroids had already been applied for several days and, thus, histologic examination did not meet the diagnostic criteria for psoriasis.

**TABLE 1:** Characteristics of CRSwNP patients with dermatologic side effects after dupilumab initiation

	Patient A	Patient B	Patient C	Patient D
Sex	Male	Female	Male	Male
Age at inclusion	30	48	36	82
Age at CRSwNP onset	19	38	20	29
Asthma	No	Yes	Yes	Yes
N‐ERD	No	Yes	Yes	No
Allergies	Dust mites	Dust mites, birch, grass pollen, artemisia, dog, cat	No	Dust mites
Pre‐existing skin disorders	No	Episodes of seborrheic dermatitis	No	Actinic keratosis
Initial dupilumab dose	300 mg every 2 weeks	300 mg every 2 weeks	300 mg every 2 weeks	300 mg every 2 weeks
Nasal polyp score (NPS)				
At inclusion (left/right)	4/4	1/3	2/1	2/3
After 6 months (left/right)	1/0	0/1	1/0	1/1
SNOT‐22				
At inclusion	33	59	28	24
After 6 months	4	3	10	NA
Dupilumab‐related side effects of the skin				
Weeks until onset of skin lesions	4	14	17	23
Clinical features	Multiple nummular, erythematous lesions with adherent scaling	Erythematous plaques with adherent scaling	Facial papules and pustules & alopecic area of the beard	Disseminated small erythematous lesions with adherent scalings
Histologic features	Psoriasiform dermatitis	Psoriasiform dermatitis	‐	Non‐diagnostic^a^ (Spongiotic dermatitis)
Dose reduction required	Yes	Yes	Yes	No
Cessation of dupilumab required	No	Yes	No	No

Abbreviations: CRSwNP, chronic rhinosinusitis with nasal polyposis; N‐ERD, non‐steroidal anti‐inflammatory drug‐exacerbated respiratory disease, SNOT‐22, sino‐nasal outcome test‐22.

^a^ Patient had received intervals of topical corticosteroids prior to histologic evaluation.

## 4 DISCUSSION

This study aimed at analyzing dermatologic side effects in 192 patients receiving dupilumab for CRSwNP in a real‐world setting. Dermatologic eruptions occurred at an incidence‐rate of 4.46 per 100PY and after a median time of 15.5 weeks after dupilumab initiation. Three of four affected patients developed a psoriasiform dermatitis.

Previous studies reported various adverse events related to dupilumab for CRSwNP occurring in phase‐II and phase‐III clinical trials. However, skin‐related adverse events (non‐injection site reactions) were not reported.^5^, ^13^ To the best of our knowledge, Swisher A.R. and co‐workers performed the only prior investigation providing data on dupilumab‐associated dermatologic side effects in CRSwNP patients. While their study included a large number of observations, no details on the nature of dermatologic eruptions were provided.^9^ The limited data available in CRSwNP patients is opposed by a multitude of publications reporting either rosacea‐like dermatitis or psoriasiform dermatitis in dupilumab‐treated patients with AD.^6^, ^10^, ^11^, ^14^ In our study, we observed one case of rosacea‐like dermatitis and three cases of psoriasiform dermatitis, with one patient additionally developing alopecia areata.

For AD, it was previously hypothesized that blocking IL‐4Rα results in inhibition of skin‐resident Th2‐mediated inflammation thereby stimulating opposing Th1 and Th‐17 pathways. This is of particular interest, since CRSwNP like AD is a classical Th2‐mediated disease. As psoriasis is a typical Th1/Th17‐mediated skin disorder, tipping the immunological scale towards a Th1/Th17 phenotype may result in activation of a psoriasis‐specific inflammatory cytokine cascade and eventually in clinical psoriasis‐like inflammation.^11^ Recently, exacerbation of rosacea caused by demodex infestation was discussed to be caused by Th1 and/or Th17 as well.^8^ We observed the development of Th1/Th17 inflammatory processes in the skin after inhibition of the Th2‐pathway by dupilumab in CRSwNP although patients had no history of type 2 associated skin disorders like AD. Our data indicates that tipping the immunological scale towards a Th1/Th17 phenotype after blocking type‐2 inflammation may induce an immunological dysbalance resulting in side effects that are not confined to the primary organ involved. First reports of AD patients developing seronegative arthropathies and enthesitis after dupilumab support our finding.^15^

Strong aspects of our study comprise that it was conducted in a real‐world setting, the longitudinal follow‐up and, importantly, the detailed patient characterization. Limitations of this study include its retrospective nature, the consecutive risk to be affected by bias and the limited number of patients.

In conclusion, dermatologic side effects in patients receiving dupilumab for CRSwNP were scarce and while the spectrum of immunologic modulation caused by dupilumab is still not entirely understood, further multidisciplinary surveillance is needed to elucidate its impact on type‐2 mediated‐ inflammation.

## CONFLICT OF INTEREST

**DC** served as a speaker and/or consultant and/or advisory board member for AbbVie, Gilead, and MSD, and received travel support from AbbVie, MSD, ViiV Healthcare and Gilead. **TB** has received personal fees as a consultant for Novartis. **FB, AT** has nothing to disclose. **TQ** served speaker and/or consultant for AbbVie, Novartis and Sanofi. TQ is a sub investigator for Abbvie, Elli Lilly, Galderma, Novartis. **JED** served as a speaker and/or consultant and/or advisory board member from Sanofi, Allergopharma, Astrazeneca, GSK and Novartis. JED is an investigator for Novartis and AstraZeneca (grants paid to her institution). **SS** served as a speaker and/or consultant and/or advisory board member from Sanofi, GSK and Novartis. SS is an investigator for Novartis and AstraZeneca (grants paid to his institution). **CB** has received personal fees as a speaker and/or consultant and/or advisory board member from AbbVie, Astra Zeneca, Bayer, Celgene, Eli Lilly, LEO Pharma, Mylan, Novartis, Pfizer, and Sanofi. CB is an investigator for Abbvie, Elli Lilly, Galderma, Novartis, Pfizer, LEO Pharma (grants paid to her institution).

## Supporting information


Appendix S1

## References

[1] Hastan D , Fokkens WJ , Bachert C , Newson RB , Bislimovska J , Bockelbrink A , et al. Chronic rhinosinusitis in Europe‐‐an underestimated disease. A GA^2^LEN study. Allergy. 2011;66:1216–23. 21605125 10.1111/j.1398-9995.2011.02646.x

[2] Jonstam K , Swanson BN , Mannent LP , Cardell LO , Tian N , Wang Y , et al. Dupilumab reduces local type 2 pro‐inflammatory biomarkers in chronic rhinosinusitis with nasal polyposis. Allergy. 2019;74:743–52. 30488542 10.1111/all.13685 PMC6590149

[3] Campion NJ , Kohler R , Ristl R , Villazala‐Merino S , Eckl‐Dorna J , Niederberger‐Leppin V . Prevalence and symptom burden of nasal polyps in a large Austrian population. J Allergy Clin Immunol Pract. 2021;9:4117–29.e2. 34265447 10.1016/j.jaip.2021.06.037

[4] Hoy SM . Dupilumab: a review in chronic rhinosinusitis with nasal polyps. Drugs. 2020;80:711–7. 32240527 10.1007/s40265-020-01298-9

[5] Bachert C , Han JK , Desrosiers M , Hellings PW , Amin N , Lee SE , et al. Efficacy and safety of dupilumab in patients with severe chronic rhinosinusitis with nasal polyps (LIBERTY NP SINUS‐24 and LIBERTY NP SINUS‐52): results from two multicentre, randomised, double‐blind, placebo‐controlled, parallel‐group phase 3 trials. Lancet. 2019;394:1638–50. 31543428 10.1016/S0140-6736(19)31881-1

[6] Quint T , Brunner PM , Sinz C , Steiner I , Ristl R , Vigl K , et al. Dupilumab for the treatment of atopic dermatitis in an Austrian cohort‐real‐life data shows rosacea‐like folliculitis. J Clin Med. 2020;9:1–11. 10.3390/jcm9041241 PMC7230957 32344789

[7] de Wijs LEM , van der Waa JD , de Jong PHP , Hijnen DJ . Acute arthritis and arthralgia as an adverse drug reaction to dupilumab. Clin Exp Dermatol. 2020;45:262–3. 31323136 10.1111/ced.14050

[8] Narla S , Silverberg JI , Simpson EL . Management of inadequate response and adverse effects to dupilumab in atopic dermatitis. J Am Acad Dermatol. 2021;86:628–36. 34126094 10.1016/j.jaad.2021.06.017

[9] Swisher AR , Kshirsagar RS , Adappa ND , Liang J . Dupilumab adverse events in nasal polyp treatment: analysis of FDA adverse event reporting system. Laryngoscope. 2021:1–7. 10.1002/lary.29992 34918342

[10] Beaziz J , Bouaziz JD , Jachiet M , Fite C , Lons‐Danic D . Dupilumab‐induced psoriasis and alopecia areata: case report and review of the literature. Ann Dermatol Venereol. 2021;148:198–201. 34175141 10.1016/j.annder.2021.02.003

[11] Flanagan KE , Pupo Wiss IM , Pathoulas JT , Walker CJ , Senna MM . Dupilumab‐induced psoriasis in a patient with atopic dermatitis and alopecia totalis: a case report and literature review. Dermatol Ther. 2022;35:e15255. 34877768 10.1111/dth.15255

[12] Brumfiel CM , Patel MH , Zirwas MJ . Development of psoriasis during treatment with dupilumab: a systematic review. J Am Acad Dermatol. 2021;86:e115. 34022319 10.1016/j.jaad.2021.05.013

[13] Bachert C , Mannent L , Naclerio RM , Mullol J , Ferguson BJ , Gevaert P , et al. Effect of subcutaneous Dupilumab on nasal polyp burden in patients with chronic sinusitis and nasal polyposis: a randomized clinical trial. JAMA. 2016;315:469–79. 26836729 10.1001/jama.2015.19330

[14] Napolitano M , Scalvenzi M , Fabbrocini G , Cinelli E , Patruno C . Occurrence of psoriasiform eruption during dupilumab therapy for adult atopic dermatitis: a case series. Dermatol Ther. 2019;32:e13142. 31664761 10.1111/dth.13142

[15] Willsmore ZN , Woolf RT , Hughes C , Menon B , Kirkham B , Smith CH , et al. Development of inflammatory arthritis and enthesitis in patients on dupilumab: a case series. Br J Dermatol. 2019;181:1068–70. 31017658 10.1111/bjd.18031

