# Supplementary material for: Dupilumab‐induced skin‐associated side effects in patients with chronic rhinosinusitis with nasal polyposis
Source: J Dermatol. 2022 Sep 30;50(1):89–93. doi: 10.1111/1346-8138.16595 (PMC10091999; doi:10.1111/1346-8138.16595)
Supplement: Supplementary file 1 — Appendix S1 [file JDE-50-89-s001.docx]

**Table-S1.** Patient characteristics

|  | Overall  N=192 | Without dermatologic side effects after dupilumab initiation  N=188 | With dermatologic side effects after dupilumab initiation  N=4 |
| --- | --- | --- | --- |
| Sex (male) | 62% (119/192) | 62% (116/188) | 75% (3/4) |
| Median age at inclusion | 47.5 (19.8) | 47.5 (19.4) | 41.8 (21.0) |
| Median age at CRSwNP onset | 31.5 (15.25) | 32.0 (15.0) | 24.5 (11.5) |
| Asthma | 65% (125/192) | 65% (122/188) | 75% (3/4) |
| Allergic rhino conjunctivitis | 53% (103/192) | 53% (99/188) | 100% (4/4) |
| N-ERD | 35% (68/192) | 35% (66/188) | 50% (2/4) |
| Median Number of precious FESS at inclusion | 2 (1) | 2 (1) | 1 (1.5) |

Abbreviations: CRSwNP, Chronic rhinosinusitis with nasal polyposis; FESS, functional endoscopic sinus surgery; N-ERD, non-steroidal anti-inflammatory drug-exacerbated respiratory disease.

**FIGURES– SUPPLEMENT**

**Figure-S1.**

**
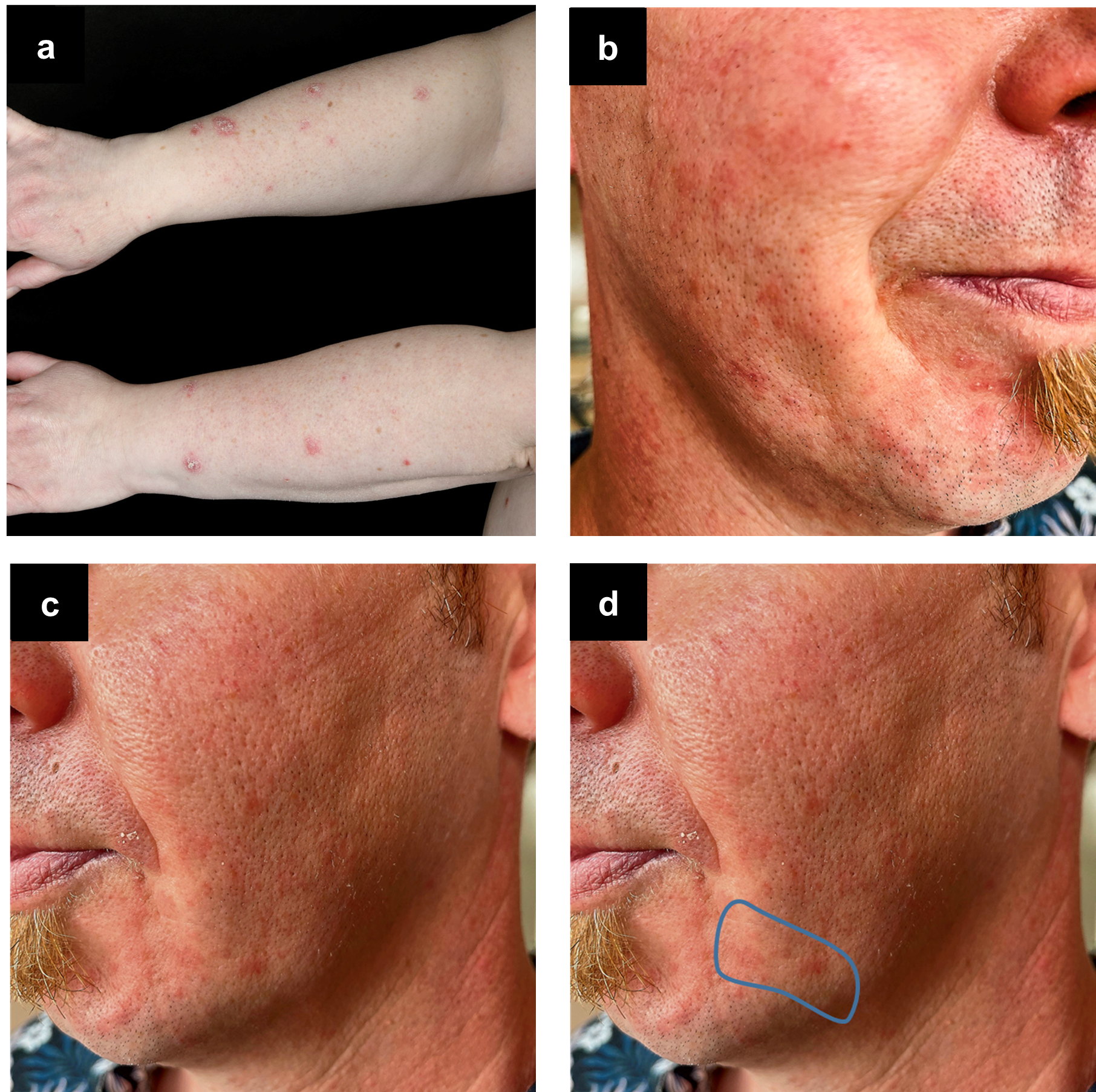
**

In patient B, erythematous plaques with distinct white scaling occurred not only on the scalp, the face and ears but also on the arms (S1a) and legs. Rosacea-like folliculitis was observed on the forehead, the cheeks, the chin and perioral area of patient C (S1b,c,d). Furthermore, patient C (S1b,c,d) developed alopecia areata affecting the beard. Since the patient shaved his beard very frequently due to the alopecia areata, it is quite difficult to identify these areas. Thus, we included a mark to aid the recognition of the alopecia areata (S1d) – there were several spots, however, the one that we highlighted is the most noticeable.
